# Influence of bone density on stability in TBW

**DOI:** 10.1186/s12891-023-07007-3

**Published:** 2023-11-15

**Authors:** Fabian R. Bischoff, Eric Tille, Franziska Beyer, Olimpiu Bota, Achim Biewener, Jörg Nowotny

**Affiliations:** 1 Klinik für Unfall-, Hand- und Orthopädische Chirurgie, Städtisches Klinikum Karlsruhe, Moltkestrasse 90, Karlsruhe, 76185 Deutschland; 2 UniversitätsCentrum für Orthopädie, Unfall- und Plastische Chirurgie, Universitätsklinikum Carl Gustav Carus Dresden, Dresden, Deutschland

**Keywords:** Tension-band wiring, Osteoporosis, Bone density, Biomechanical testing, Osteosynthesis

## Abstract

Osteoporosis is a common disease that leads to a reduction in bone density and increases the risk of fractures. Stable surgical treatment is particularly important for these fractures. The aim of this study was to examine the influence of bone density in the area of ​​the proximal ulna on the failure of the fixation technique of K-wires in tension band wiring (TBW). We provided 10 ulna specimens with TBW and biomechanically examined the pull-out strength of bi- and tricortical K-wires. Bone density measurement was performed using qCT. In the paired t-test, the tricortical group showed a significantly higher pull-out strength in relation to bone density than the bicortical group (*p* = 0.001). Furthermore, the Pearson correlation showed a high influence of bone density on pull-out strength in the tricortical group (*r* = 0.544), but without significance (*p* = 0.100).

Our work shows that bone density has a direct effect on the pull-out strength of K-wires in TBW. TBW should therefore be used as osteosynthesis technique, especially in young patients with non-osteoporotic bones. In the case of osteoporotic fractures, alternative procedures should be preferred.

## Summary

Osteoporosis is a common disease. We examined the influence of bone density on the pull-out strength of K-wires in TBW at the olecranon. We were able to show that the bone density has a major influence on the pull-out strength and recommend other osteosynthesis techniques for fractures in osteoporotic bones.

## Introduction

According to the definition of the World Health Organization (WHO), osteoporosis is a systemic skeletal disease which is characterized by a reduction of bone mass of more than two standard deviations compared to a reference group (middle-aged white women) [1]. According to the International Osteoporosis Foundation (IOF), 32 million people over the age of 50 years suffered from osteoporosis in Europe in 2019. This accounts for approximately 5.6% of the European population aged 50+ [2]. On average, 8.5% of German woman and men aged 50–79 years have been diagnosed osteoporosis, whereby women have a much higher proportion of 13.1%. Furthermore, the lifetime prevalence in women increases significantly with age. A lifetime prevalence of around 3.2–3.3% is observed, while it is 4.1% at the age of 50–59 years and rises to 25.2% in the age group 70–79 years [3].

In the 2017 guideline for the diagnosis of osteoporosis, the German osteology association (DVO) describes the DEXA scan (Dual Energy X-Ray Absorption) as the gold standard for osteodensitometry [4]. With this method, the attenuation of two x-rays with different energy levels transmitted through the bones is determined and compared with standard values. Quantitative computed tomography (qCT) is available as an alternative procedure. In this cross-sectional imaging, the intensity of the attenuation of X-rays transmitting through bone structures is determined and compared with standard values. The advantage is the more precise resolution and therefore better differentiation between cortical and spongiosa bone [4]. In recent years quantitative ultrasound methods have been described as an alternative for osteodensitometry diagnostics. The radiofrequency echographic multi spectrometry method (REMS) has already demonstrated diagnostic reliability comparable to DEXA in studies. With the REMS method, the ratio of resorbed and reflected ultrasound waves is compared and set in relation to standard values. The advantage of this method is the lack of radiation exposure [5].

Fractures in osteoporotic patients occur frequently and are considered as diagnostic criteria for manifest osteoporosis. Fractures of the proximal femur, the distal radius and vertebral body [6], as well as proximal humerus [7] are the most common. These fractures require a stable osteosynthesis, as there is an increased rate of osteosynthesis failure in osteoporotic fracture treatment [8, 9]. Stable osteosynthesis methods include locking plate osteosynthesis and intramedullary nailing, which may be supported by bone augmentation (e.g. with allografts and autografts, bone cement or bone grafts) [10, 11].

The aim of this study was to examine the influence of the bone density in the area of ​​the proximal ulna on the failure of the fixation technique of K-wires in tension band wiring (TBW).

## Methods

### Specimens

A total of 10 ulna specimens were examined after receipt from the Institute for Anatomy at the University of Dresden. During the preparation, no macroscopic malignancies and other structural pathologies and injuries of the examined bone could be detected. The arms were stored in freezers (Liebherr Type 40073 1, Germany) at -22 °C until preparation and in a refrigerator at 4 °C (Liebherr glassline, Germany) during the preparation process. The soft tissue was completely removed. The ulnae were then cut 15 cm measured from the proximal end. A transverse intraarticular osteotomy was performed on the proximal ulna according a Schatzker A - respectively AO 2U1B1 fracture. In the proximal end, 2 K-wires were inserted parallel in the usual way of tension band wiring. Finally, the distal end of the ulna was cast in dental cement (Excalibur type 4 golden brown, LOT 16.1254) in order to obtain a stable construction for testing.

### Bone density measurement using quantitative CT

QCT (Siemens, Munich) was used to measure bone density. A three-dimensional measurement was performed after calibration with a bone density phantom. The layer thickness was 0.75 mm, the rotation time 180 mAs (product of rotation time and tube current) and the tube voltage amounted 80 kV. The volume-related dose index (CTDI) was 4.53 vol*mGy.The evaluation of the qCT images was performed with the computer program AGFA IMPAX EE (Version: R20XVIISU3). The average bone density at the level of the proximal radioulnar joint was determined.

### Implants

**Fig. 1:**
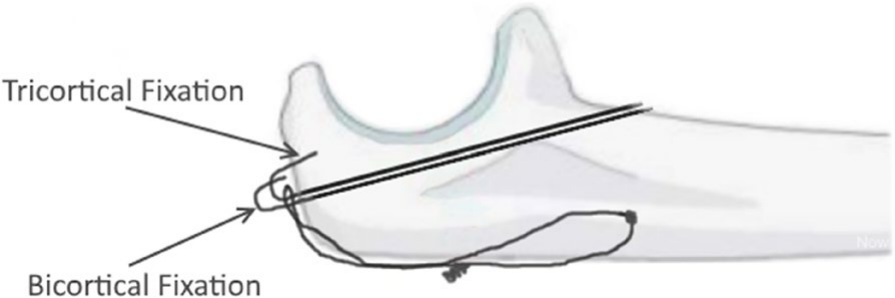
Schematic drawing of the bi- and tri-cortical fixation in TBW. (With permission of Nowotny et al. [12])

For the biomechanical testing we used K-wires with a diameter of 1.8 mm (Aesculap, Fa. Braun, Germany). Attention was paid to correct positioning and parallelism. The K-wires were inserted tangentially below the joint surface with an angle of 20° until they perforated the anterior cortex of the ulna. As it is usual in TBW the K-wires were attached more radially to avoid a possible damage of the ulnar nerve. Using a randomization list, one K-wire was inserted into the proximal ulna on the radial side in either bicortical or tricortical fixation technique (Fig. 1), while the other fixation technique was used on the ulnar side.

### Test setup

**Fig. 2:**
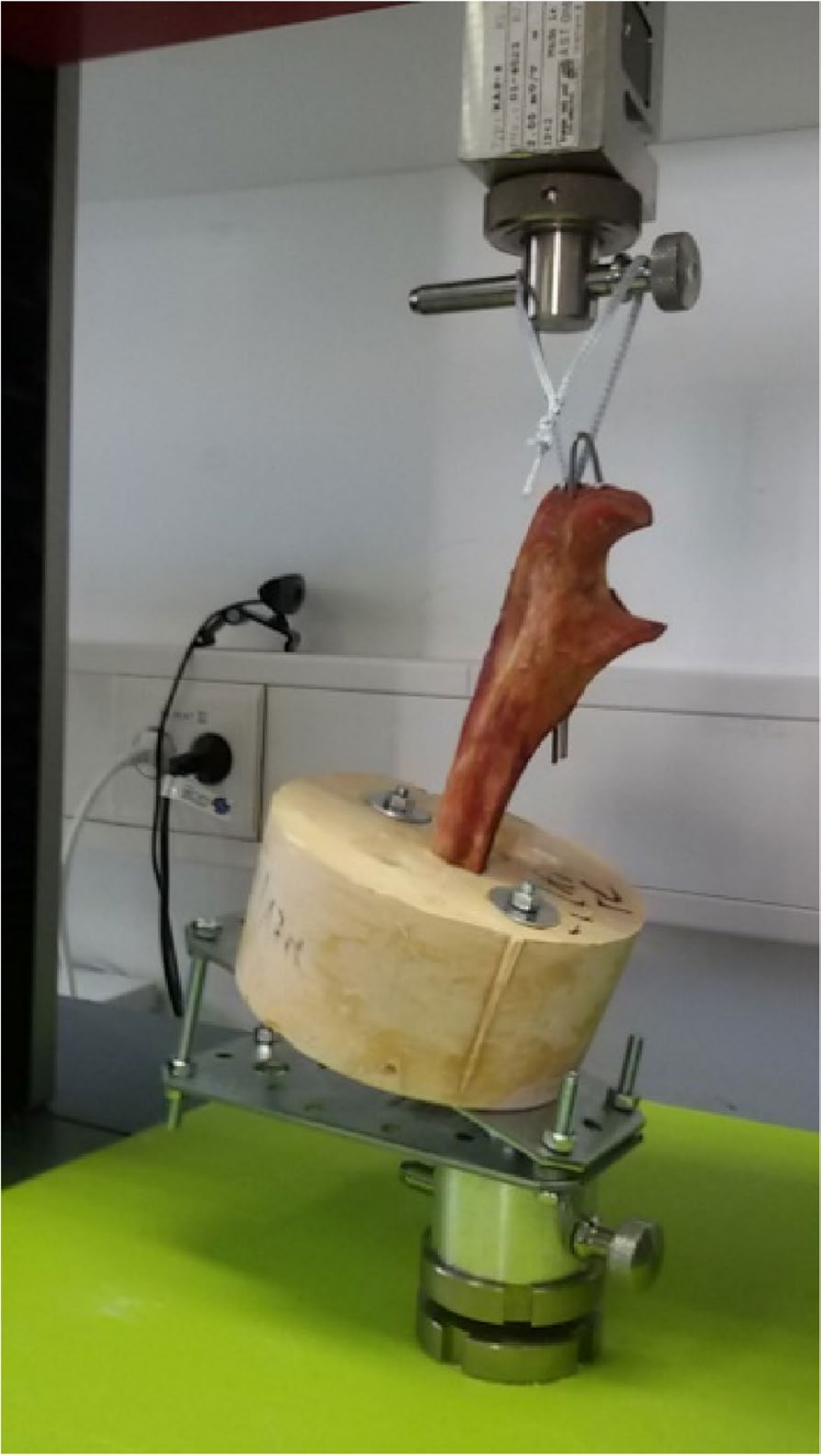
Presentation of the biomechanical test setup

The tests were carried out using a Zwick/Roell® material testing machine (Type Z010, BT1-FB010TN.D30, Zwick GmbH, Ulm, Germany). The ulnae were attached to a custom-made construction with the cement foot positioned exactly under the 10 kN load cell in order to ensure an optimal position of the wires (Fig. 2) for a linear pullout setup. A highly cross-linked FiberTape® was used for the connection between the K-wires and the traction device and load cell. The material testing machine was set with a preload of 1N and the pull-out test was started with a speed of 10 mm per minute until failure. The tests were performed as a linear pullout test of the K-wires. Tricortical and bicortical measurements were performed sequentially on each ulna. Loosening of the wire was defined as the end point of the tests. All tests were successfully completed. No failure of the experimental setup or components was observed. The statistical evaluation was performed with SPSS (statistic software, version 24, IBM, NY, USA). A simple paired t-test and Pearson correlation was used. The level of significance was set at *p* < 0.05.

## Results

The load to failure data has already been published in a previous publication [12]. The pull-out strength in relation to bone density describes how much force per Hounsfield Unit (HU) was required to loosen the K-wire from the bone. For the bicortical group, the mean value was 0.47 N/HU (min. 0.25 N/HU, max. 0.83 N/HU, SD 0.20 N/HU). In the tricortical group, the value was 0.58 N/HU (min. 0.42 N/HU, max. 0.90 N/HU, SD 0.17 N/HU). In the paired t-test, the tricortical group showed a significantly higher pull-out strength per HU (*p* = 0.001).

**Fig. 3:**
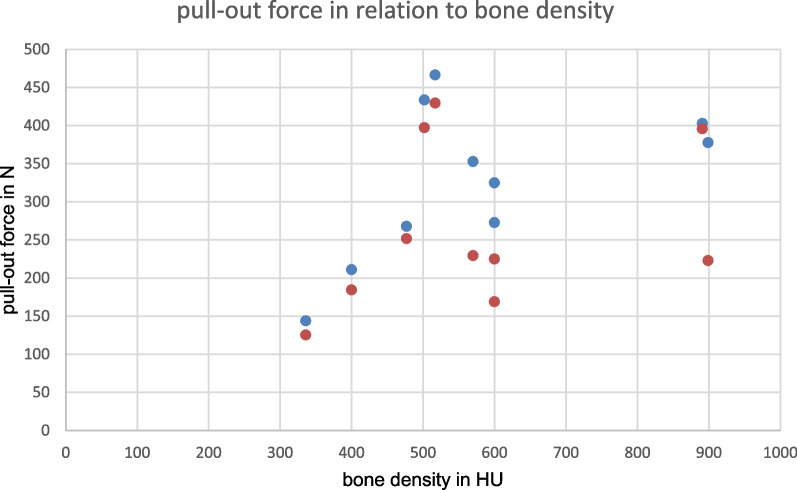
Pull-out force (N) in relation to bone density (HU) (red points = bicortical group, blue points = tricortical group).

Figure 3 shows the pull-out force compared to bone density. Table 1 shows an overview of all test results.

**Table 1:** Overview of the results

Preparationnumber	Sex	Age (years)	F max. bicortikal(N)	F max. tricortikal(N)	BMD (HU)	N/HU tricortikal	N/HU bicortikal
7/0.17 right	female	62	395.7	402.5	891	0.4517	0.4441
7/0.17 left	female	62	222.7	377.4	899	0.4198	0.2477
20/17 right	male	83	397.1	433.5	502	0.8635	0.791
23/17 left	male	76	429.4	466.2	517	0.9017	0.8306
24/17 left	male	91	184.3	210.9	400	0.5273	0.4608
24/17 right	male	91	251.6	267.7	477	0.5612	0.5275
25/17 right	female	83	229.3	352.7	570	0.6188	0.4023
25/17 left	female	83	224.9	324.8	600	0.5413	0.3748
31/15 right	female	92	168.8	272.6	600	0.4543	0.2813
31/15 left	female	92	125.3	143.7	336	0.4277	0.3729
Average		81.5	263	325	688	0.58	0.47
SD		11.5	106	102	170.3	0.17	0.2

The Pearson correlation shows a large effect of bone density on the pull-out strength for the tricortical group with an *r* = 0.544, but without significance (*p* = 0.100). The Pearson correlation shows a medium effect of bone density on the pull-out strength for the bicortical group with an *r* = 0.304, but also without significance (*p* = 0.390)

## Discussion

The aim of this study was to investigate the influence of bone mineral density on strength of K-wires in TBW in a demographically steadily aging society. Therefore we decided to perform a linear pullout test of K-wires which were inserted in the typical way during TBW procedure in the proximal ulna. Although a nonlinear pullout would represent a more the physiological setup, the influence of the bone mineral density on the K-wire strength would not be in the focus. So we decided neither to simulate the influence of dynamic triceps tendon force nor the static pressure of the humerus.

To our knowledge, no study has evaluated the relation between bone density and pull-out strength of K-wires in TBW yet. Our study demonstrated for the first time that bone density has an effect on the stability of K-wires in tension band wiring at the olecranon. As can be seen in Fig. 3, the wire strength in the bone increases with higher bone density. Pearson correlation revealed a large effect of bone density on pull-out strength for the tricortical group, but without significance. This was presumably due to the low number (*N* = 10) of available bone donors and has already been observed in other biomechanical studies [13].

Nevertheless, with *p* = 0.100, at least a statistical trend can be presumed. For the bicortical group, the Pearson correlation shows a medium effect of bone density on the pull-out strength, also not significant with *p* = 0.390. Although the correlations were not significant, the tricortical group showed a greater effect of bone density on pull-out strength.

Since we only obtained the arms of donors, we decided to investigate bone mineral density using the common technique of qCT. Thus, conventional methods of bone mineral density measuring such as Dual energy X-ray absorptiometry (DXA) or quantitative ultrasound (QUS) could not be used. However, qCT is an established procedure to determining bone mineral density [14]. Melton et al. showed that bone density decreases with increasing age in both women and men and that reduced bone density increases the risk of fractures [15]. There are several prediction tools in the literature for estimating the fracture risk in the context of osteoporosis. Age has been found as a central factor in each of these prediction tools [16]. Kirilova et al. investigated the predictive power of the osteoporosis self-assessment tool (OST) [OST value = (body weight-patient age)*0.2] for the risk of developing osteoporosis [17]. In addition to patient weight, age plays an important role in the OST. Kirilova et al. calculated the risk for patients using OST and compared these with the bone density values ​​determined using DEXA. They observed that 95.5% of the calculated high-risk patients also showed radiological evidence of osteoporosis and therefore emphasized that patient’s age is an important factor for the risk of osteoporosis. The mean age for the high-risk group was determined by Kirilova et al. at 76 years, which is lower compared to the average age of our body donors [17]. This suggests that our body donor collective had an increased risk of osteoporosis and therefore a reduced bone density was expected. In 2005, Todisco and Trisi evaluated the relation between bone density and the histomorphological composition of bone [18]. They used computed tomography (CT) to examine the jaw bones in 23 patients who were to receive dental implants. The study evaluated the bone density in the implant region in HU. They also used a bone density phantom for calibration. During the implantation of the dental implants, they took small cylindrical samples and compared the histological density with HU recorded by CT for the same sites. Todisco and Trisi showed in their publication that there is a statistically significant correlation between measurements of HU by CT and histomorphological density examinations [18]. While the examination was carried out in a different part of the body, it is nonetheless probable that the results can also be valid in other regions, such as the olecranon. Thus, the HU recorded in our CT examinations can be compared with the actual bone density.

The average bone density in our investigation was 579 HU. Gruszka et al. examined biomechanically the resilience of a tension band plate in comparison to TBW. They quantified the bone density with an average of 671 HU [19], which is above our measurements. This could be mainly due to the fact that the body donors in our study were older than the collective in the publication of Gruszka et al. (average of 67 years).

As a further point, our results show that a bone density above 570 HU increases the necessary pull-out strength by almost 100 N and thus an enormous gain of stability can be expected (Fig. 3). This could be due to the fact that, with rising bone density, there is an increase in the number of trabeculae at the histological level, increasing the friction of the K-wire in the bone structure [18].

Halvorson et al. studied the effect of bone density in pedicle screw fixation. They evaluated the maximum pull-out strength and showed that there is a high correlation with the bone density [20]. The study results are not directly comparable due the fact that they investigated a different body region and the bone structure of a vertebral body does not correlate with the ulnar bone structure. Nevertheless, the results lead to a similar trend as seen in our study. Furthermore, Chapman et al. evaluated the factors influencing the pull-out strength of spongiosa screws. For this purpose, they used a unicellular polyurethane sponge with three different densities, each close to or in the range of the comparable bone density of spongiosa. Their data indicated that a higher material density also requires higher pull-out strength [21]. This study however cannot be directly compared to our study, since both, the medium and the material that was examined, differ from our experimental conditions. Nevertheless, the results of Chapman et al. can be interpreted in a similar way, compared to the current investigation. In the biomechanical study of Amirouche et al. the focus was on the relation between bone density and the insertion angle of pedicle screws. As Chapman et al., they also used a polyurethane sponge model with different densities to depict the different densities of the bone. They observed a high correlation between bone density and tensile strength. Especially with increasing bone density, an influence on the pull-out strength in the different insertion angles of the pedicle screws was seen [22]. Seebeck et al. tested the effect of cortical thickness and bone density of spongiosa on the load-bearing capacity of screws during internal fixation. They compared the insertion of screws in plate fixation and intramedullary nailing and described that the cortical thickness and the spongiosa density had a significant influence on the load capacity of screws [23]. This also coincides with our results that the bone quality has an influence on the stability and tensile strength of the osteosynthesis material.

## Conclusion

Bone mineral density has an influence on every osteosynthesis technique. In particular bone density has a direct effect on the pull-out strength of K-wires in TBW. Especially in patients with low bone mineral, the primary stability of the chosen osteosynthesis technique should be considered. TBW should therefore be used as osteosynthesis technique, especially in young patients with non-osteoporotic bones. In the case of osteoporotic fractures, alternative procedures (e.g. locking plate fixation) should be preferred.

## Data Availability

The material and the data are available.

## Declarations

Written informed consent was obtained from all individual participants included in the study.

All authors confirm that this study adhered to the Declaration of Helsinki.
